# Clinical application of 4% sodium citrate and heparin in the locking of central venous catheters (excluding dialysis catheters) in intensive care unit patients: A pragmatic randomized controlled trial

**DOI:** 10.1371/journal.pone.0288117

**Published:** 2023-07-03

**Authors:** Yuchun Deng, Jie Xing, Zhi Tan, Xiaohua Ai, Yi Li, Liqin Zhang

**Affiliations:** 1 Intensive Care Unit, People’s Hospital of Zhongjiang County, Zhongjiang County, Sichuan Province, China; 2 School of materials science and engineering, Sichuan University, Chengdu, China; Sant Anna Hospital: Clinica Sant’Anna, SWITZERLAND

## Abstract

**Objectives:**

The feasibility of utilizing 4% sodium citrate as an alternative locking solution for central venous catheters (CVCs) (excluding dialysis catheters) was assessed.

**Methods:**

Using heparin saline and 4% sodium citrate as locking solution, then 152 patients in ICU undergoing infusion with central venous catheters, were randomly assigned to receive either 10 U/mL heparin saline or 4% sodium citrate. The used outcome indicators include: four indexes of blood coagulation at 10 minutes after locking and 7 d after the first locking, bleeding around the puncture site and subcutaneous hematoma rate, gastrointestinal bleeding rate, catheter indwelling time, catheter occlusion rate, catheter-related bloodstream infection (CRBSI) rate, rate of ionized calcium < 1.0 mmol/L. The main outcome indicator was the activated partial thromboplastin time (APTT) at 10 min after tube locking. The trial was approved by relevant authorities (Chinese Clinical Trial Registry, no: ChiCTR2200056615, registered on February 9, 2022, http://www.chictr.org.cn; Ethics Committee of People’s Hospital of Zhongjiang County, no: JLS-2021-034, approved at May 10, 2021, and no: JLS-2022-027, approved at May 30, 2022).

**Results:**

Among the main outcome measures, the heparin group showed a significant increase in APTT compared to the sodium citrate group at 10 min after locking (LSMD = 8.15, 95%Cl 7.1 to 9.2, *P* < 0.001). Among the secondary outcome measures, the heparin group demonstrated a significant increase in prothrombin time (PT) compared to the sodium citrate group at 10 minutes after locking (LSMD = 0.86, 95%CI 0.12 to 1.61, *P* = 0.024). It is found that APTT (LSMD = 8.05, 95%CI 6.71 to 9.4, *P* < 0.001), PT (LSMD = 0.78, 95%CI 0.14 to 1.42, *P* = 0.017) and fibrinogen (FB) (LSMD = 1.15, 95%CI 0.23 to 2.08, *P* = 0.014) at 7 d after locking are increased in the heparin group compared to sodium citrate group. There was no significant difference in catheter indwelling time between the two groups (*P* = 0.456). The incidence of catheter blockage was lower in sodium citrate group (RR = 0.36, 95%CI 0.15 to 0.87, *P* = 0.024). No CRBSI occurred in the two groups. Among the safety evaluation indexes, the incidence of bleeding around the puncture site and subcutaneous hematoma was lower in sodium citrate group (RR = 0.1, 95%CI 0.01 to 0.77, *P* = 0.027). There was no significant difference in the incidence of calcium ion < 1.0 mmol/L between the two groups (*P* = 0.333).

**Conclusions:**

In ICU patients using CVCs (excluding dialysis catheters) infusion, employing 4% sodium citrate as a locking liquid can reduce the risk of bleeding and catheter occlusion without any hypocalcemia.

## 1. Introduction

Central venous catheters (CVCs) in adults are inserted in the subclavian, jugular, or femoral vein, with the catheter tip positioned within the superior or inferior vena cava [1]. These CVCs serve as crucial tools in modern critical care medicine, playing a pivotal role in a wide range of clinical applications. They are extensively utilized for hemodynamic monitoring, blood purification, administration of blood products and medications, as well as the delivery of total parenteral nutrition [2–4]. The utilization of CVCs results in reduced hospitalization durations, enhanced safety measures, and decreased healthcare costs [3]. However, in the case of catheter-related bloodstream infection (CRBSI) [5–7], catheter occlusion [2], bleeding complications in CVCs [8], it will not only affect the therapeutic effect of the primary disease, prolong the hospital stay, and increase the mortality of patients, but also cause the waste of medical resources and increase hospitalization costs [2, 6, 9, 10]. For continuous and effective use of CVCs, catheter locking technology is an important step to ensure smooth catheter and effective prevention of thrombosis, bleeding complications, and CRBSI.

According to the Infusion Nurses Society, 0–10 U/mL heparin saline is recommended as CVC locking fluid for ICU patients [11]. In China, it is generally used 10 U/mL of heparin saline to lock the infusion CVCs for adults [12] and used normal saline (0.9% sodium chloride) to lock the infusion CVCs for children [13]. However, as a tube locking solution, heparin is found to have many disadvantages [2, 8], such as (i) causing systemic anticoagulation, especially in the intensive care unit and some high-risk patients (severe sepsis patients, postoperative patients, etc.) with increased bleeding risk [14]; (ii) triggering heparin-induced thrombocytopenia (HIT) [15–17]; (iii) interfered by the level of antithrombin III, and the low level of antithrombin III commonly seen in ICU patients limiting the anticoagulant effect of heparin; (iv) promoting the formation of staphylococcus aureus biofilm in a dose-dependent manner [18], giving rise to increased CRBSI and catheter obstruction [19–21].

To reduce the risks of bleeding and HIT, normal saline (0.9% sodium chloride) is suggested to replace heparin as the locking fluid of CVCs [3, 4, 22, 23]. However, normal saline (0.9% sodium chloride) does not possess an anticoagulation effect, which may increase the occlusion rate of CVCs. A meta-analysis of 10 studies with 1672 participants showed that heparin had a lower occlusion rate than that of normal saline (RR = 0.7, 95%CI 0.51–0.95, *P* = 0.02) [24]. Other researchers have proposed the use of antibiotic locking fluid to reduce CRBSI, however, this practice may promote the generation of resistant bacteria [25, 26]. Hence, looking for a safe alternative to heparin is urgent to improve the above situations.

Sodium citrate tube locking liquid has local anticoagulant property and hence it has no effect on coagulation function [27–30]. As a tube locking fluid with a high antibacterial concentration, sodium citrate can inhibit the growth of staphylococcus aureus and staphylococcus epidermidis by preventing the formation of microbial biofilms, thus reducing catheter occlusion and CRBSI without the generation of bacterial resistance [20, 31, 32]. According to international guidelines regarding dialysis catheter blocking systems, 4% sodium citrate has been identified as the most cost-effective and safe option [21].

Currently, numerous studies have been conducted to compare the effects of sodium citrate locking solution and heparin locking solution on central venous catheter (CVC) locking in hemodialysis patients. These studies consistently indicate that sodium citrate, as a catheter locking fluid, exhibits superior effectiveness and safety compared to heparin [8, 9, 29, 30, 32–37]. However, the use of sodium citrate as a locking for CVCs (excluding dialysis catheters) in ICU patients is not involved in previous studies. Because of the differences between the two catheters (e.g., different catheter lengths, diameters, and uses), it is inappropriate to extrapolate the results of hemodialysis CVCs to non-hemodialysis CVCs. Sodium citrate is also advocated to use in CVCs (excluding dialysis catheters) [38].

Therefore, we conducted a pragmatic randomized controlled trial in which ICU patients were enrolled to compare the efficacy of 10 U/mL heparin saline and 4% sodium citrate in CVCs (excluding dialysis catheters) catheter locking from two aspects of therapeutic efficacy and safety.

## 2. Material and method

### 2.1. Study design

This study is a prospective, three-blind, randomized, parallel grouping, standard control, single-center clinical trial conducted according to the reporting specifications of randomized controlled trials (CONSORT). The checklist can be seen in S1 File. Details of the research process are listed in S2 File.

### 2.2. Study participants

The first patient is enrolled on 31st December 2021 and the last patient is enrolled on 24th July 2022. The last observation is collected on 31st July 2022. We state that our CT registration was later that the date of enrolling the first patient because of the delay caused by the COVID-19 pandemic. Our CT registration actually was first submitted before December 31st, 2021, but it was not approved until February 9th, 2022 due to the extended review time of the website during the pandemic. Despite the CT registration being late, the trial still was conducted according to the original trial plan. (Chinese Clinical Trial Registry, no: ChiCTR2200056615, http://www.chictr.org.cn.)

For real-world research purposes, we relaxed the criteria and did not exclude patients on anticoagulants (Including patients treated with continuous renal replacement therapy and treated with enoxaparin). Patients aged 18 to 80 years who received CVCs infusion during their ICU stay were enrolled. We excluded women during pregnancy, perinatal or lactation, and patients with heparin or sodium citrate allergy or coagulation abnormalities. If patients withdraw their informed consent, are removed from the ICU, die, or have a catheter removed, the study is considered to be discontinued. The first placement of CVCs in each patient was recorded.

### 2.3. Randomization

The experimental group and the control group are randomly divided into 1:1. Random method: Random numbers are generated using SPSS 25.0 statistical software by the center’s personnel in charge of randomization, and group by the rank of the random number and group principle. Opaque envelopes are used to lock the results, and the envelopes are numbered. When a patient needs CVCs, the envelope is opened in numerical order to determine whether the patient is admitted to the experimental or control group. The allocation order is hidden until the end of the trial.

### 2.4. Masking

In this study, 2 nurses in the venous configuration center are responsible for marking test numbers and preparing locking fluids. No further cover-up is required because both types of locking fluids are colorless and odorless. The prepared locking solution is distributed to highly skilled and experienced ICU nurses who specialize in critical care. These nurses undertake the responsibility of performing the catheter locking procedure with utmost proficiency and expertise. Two additional investigators are tasked with the rigorous observation and systematic collection of clinical data. It is imperative that both investigators independently confirm the positive results (catheter occlusion, bleeding around the puncture site and subcutaneous hematoma) to ensure their validity and reliability. During the study, only the 2 nurses who prepare the tube-locking solution knew about the grouping situation, while the subjects, operators, investigators, and statisticians are all unaware of the grouping situations (S2 File).

### 2.5. Interventions

To ensure the reliability and authenticity of the experiments and reduce the bias caused by external factors, centralized training is conducted for the general nurses in the intensive care unit who participate in the research to achieve the purpose of homogenization.

Selection of locking fluid and locking method: The selected subjects are all indwelled double-lumen catheters with single-use CVCs kit TYPE I (Henan Tuoren Medical Instrument Group Co., LTD.); specifications: 2-7FR-20cm, outer diameter of catheter 2.4 mm, length of catheter 20 cm. All catheters are inserted under ultrasound guidance and standard hygiene precautions are taken to minimize contamination.

- Experimental group: Taking 5 mL from a dose of 200 mL of 4% sodium citrate (Chengdu Qingshan Likang Pharmaceutical Co., LTD., Production batch NO. A2101013) as tube locking liquid.
- Control group: Taking 0.4 mL from a dose of 12500 u/2mL Heparin sodium injection (Chengdu Haitong Pharmaceutical Co., LTD., Approval no. H51021209) and adding 250 mL 0.9% sodium chloride injection as a mixture. After uniform preparation, a 5 mL mixture is taken as the tube locking liquid. The concentration of heparin in tube locking fluid is 10 U/mL.

Flushing CVCs must be performed using turbulence with 10 mL 0.9% sodium chloride injection before and after each infusion, medication, parenteral nutrition, transfusion of blood products, and replacement of pipeline equipment. Sodium citrate tube locking solution is used in the experimental group, heparin tube locking solution is used in the control group and a positive-pressure locking technique is used to lock the tube. CVCs that are not currently in use are flushed and locked once a day [12, 39–42], suctioning out the locking solution when using CVCs. During the patient’s ICU stay, the researchers conducted daily interviews with the nurses performing catheter locking procedures at the bedside. In the case of positive findings, such as catheter occlusion, bleeding around the puncture site and subcutaneous hematoma, a secondary confirmation was undertaken to ensure accuracy. These validated data results were meticulously documented in the patient’s medical records. Flushing and locking procedures are detailed in S3 File.

### 2.6. Outcome measure

Main outcome indicator: activated partial thromboplastin time (APTT) at 10 min after tube locking. Secondary outcome indicators: APTT at 7 d after initial tube locking, prothrombin time (PT) at 10 min after tube locking and 7 d after first tube locking, international normalized ratio (INR), fibrinogen (FIB), thrombin time (TT), catheter indwelling time, catheter occlusion rate, and CRBSI rate. Safety evaluation index: bleeding around the puncture site and subcutaneous hematoma rate, gastrointestinal bleeding rate, rate of ionized calcium < 1.0 mmol/L.

Judgment criteria are as follows. Four indexes of blood coagulation include the blood of patients was drawn by venipuncture of peripheral blood before tube locking, 10 minutes after tube locking, and 7 d after the first tube locking, respectively, then comparing APTT, PT, INR, FIB, and TT. Catheter occlusion standard: (i) Partial occlusion. Infusion can be performed but blood cannot be withdrawn, the infusion rate is slowed down, local pain is present, there is resistance to flushing, or exudation occurs. (ii) Complete occlusion. It cannot be infused, withdrawn blood, or is unable to flush. When any of the above occurs, it is indicated by catheter occlusion [38, 43]. CRBSI is determined in accordance with clinical practice guidelines of the Infectious Diseases Society of America (IDSA), as follows: (i) One semi-quantitative catheter culture is positive (≥15 CFU per catheter segment) or quantitative catheter culture is positive (≥1000 CFU per catheter segment), while the peripheral venous blood is also positive, and the same microorganism appears as the catheter segment. (ii) Blood is collected from the catheter and peripheral vein simultaneously for quantitative blood culture, and the colony count ratio (catheter blood: peripheral blood) of the two is ≥ 5:1. (iii) Blood samples are collected from both the central venous catheter and peripheral vein for qualitative blood culture. The appearance-positive time of central venous catheter blood culture is at least 2 h earlier than that of peripheral blood culture. (iv) The culture of peripheral blood and pus at the outlet of the catheter are both positive and of the same strain of microorganism. The occurrence of any one of the above items can be proved as CRBSI [6, 44]. Bleeding around the puncture site: nurses assessed CVCs at shift shifts to determine whether there is blood exudation in the dressing. Subcutaneous hematoma: hematoma or bruising around the place of catheterization is existed. Digestive tract bleeding: gastric juice occult blood test positive, or fecal occult blood test positive stool, and one of both occurrences is judged to be digestive tract bleeding.

### 2.7. Statistical analysis

Continuous variables are tested by histogram and Shapiro-Wilk test, and those that conformed to normal distribution are expressed as mean and standard deviation (SD); those that did not conform to normal distribution are expressed as median and quartile spacing. By performing covariance adjustment on the four indexes of blood coagulation prior to locking, the least-squares mean difference (LSMD) and its corresponding 95% confidence interval (95%CI) were computed. When the proportion of missing values was below 20% and the assumption of random missingness was made, multiple imputation by chained equations (MICE) was utilized to handle the missing variables. Specifically, predictive mean matching was employed to impute the missing values of the four indexes of blood coagulation 7 d after the initial locking [45]. Furthermore, a sensitivity analysis was performed to assess the robustness of the imputed results. The Wilcoxon Mann-Whitney test is used for those that do not conform to the normal distribution. Categorical data are presented in frequencies and percentages, and the Chi-square test or Fisher’s exact test is used. The occurrence rates of various indicators were calculated using modified Poisson regression analysis to obtain the relative risks (RR) and their corresponding 95%CI (catheter occlusion rate, bleeding around the puncture site and subcutaneous hematoma rate , and rate of ionized calcium < 1.0 mmol/L) [46]. All statistical analyses are based on two-sided hypothesis testing, with α = 0.05 as the test level, and *P* ≤ 0.05 as statistically significant.

Due to the uneven distribution of the catheter insertion location as baseline data, we performed a post hoc analysis to adjust this confounding factor: using the internal jugular vein and subclavian vein as subgroups, an analysis of covariance is performed in the four indexes of blood coagulation 10 minutes after locking, and a forest map is drawn. The α segmentation is used to control class I errors that come from multiple comparisons, and *P* ≤ 0.025 is considered to be statistically significant.

To get the following data, before the start of the experiment, a two-month pre-experiment is conducted. The results show that the mean APTT of the control group is 36.74±4.74 seconds, and the mean APTT of the experimental group is 32.84±7 seconds. Setting two-sided α = 0.05 and the power is 90%, the sample size of the experimental group N1 = 51 cases and the sample size of the control group N2 = 51 cases are calculated by PASS 15 software. Taking into account the 20% loss to follow-up and refusal to follow-up, at least 64 subjects in the experimental group and 64 subjects in the control group are finally required, and a total of at least 128 cases are included. This study includes 152 subjects totally. Sample size calculation was only conducted for the main outcome indicator, namely the APTT at 10 min after tube locking. The lack of sample size calculation for the remaining outcome measures may have led to inadequate statistical power and an increased risk of type II error, thereby potentially yielding false-negative results.

All data are analyzed using the IBM SPSS Statistics for Windows (SPSS, ver. 25.0, USA) and R software (version 4.2.1, available at https://www.r-project.org/).

### 2.8. Ethics approval and consent to participate

This study has been reviewed and approved by the medical ethics committee and the academic committee of Zhongjiang County People’s Hospital (ethics approval number: JLS-2021-034, date of approval May 10, 2021; JLS-2022-027, date of approval May 30, 2022; medical research record number: MR-51-21-014013). Since the patient is in a critical condition and has no ability to sign the consent form, the patient’s close relatives (parents, children, siblings) act as agents to sign the informed consent form. The ethics committee was aware of and approved this consent procedure. The trial was conducted in agreement with the updated Declaration of Helsinki.

## 3. Results

### 3.1. Patient population

**Fig 1:**
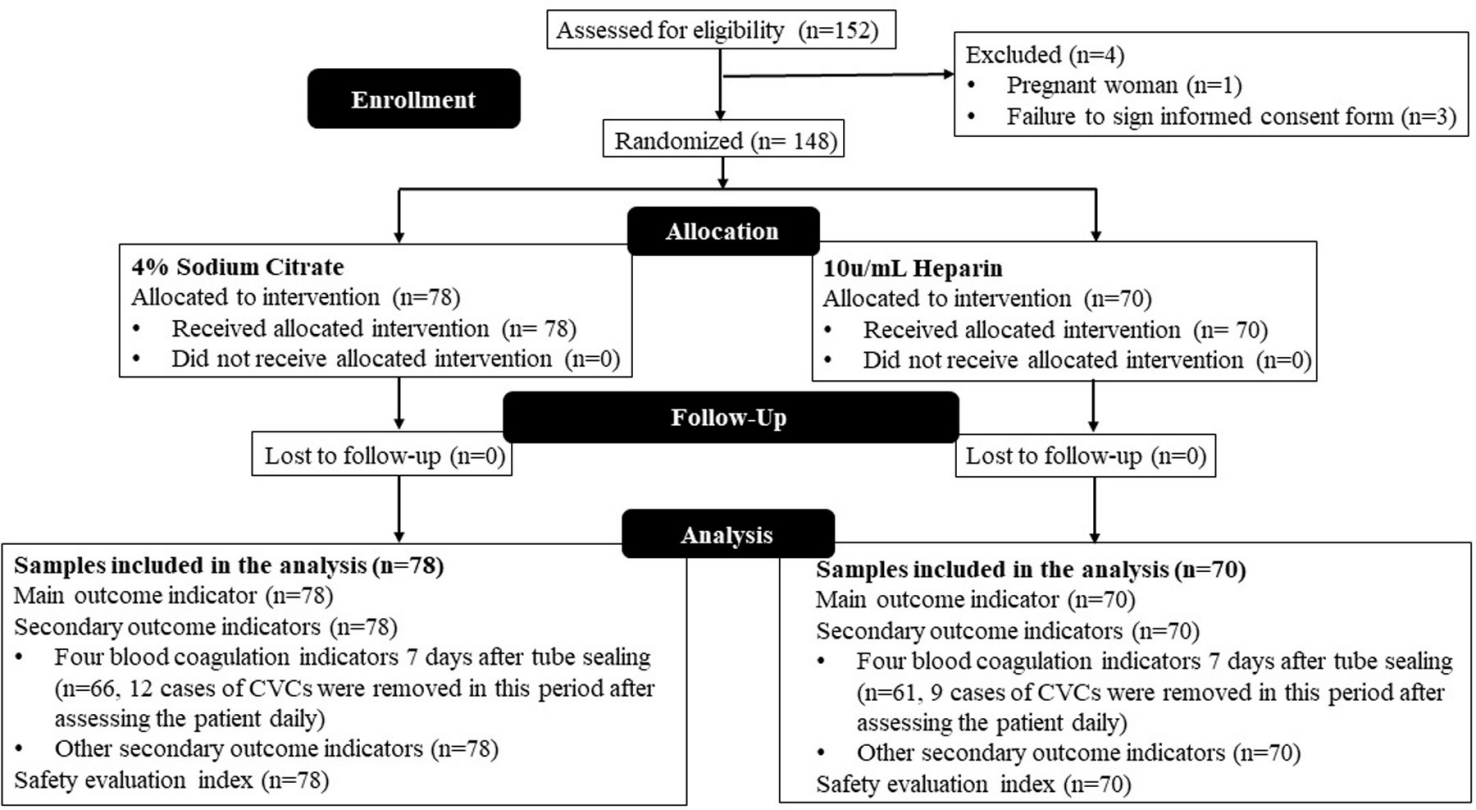
CONSORT flowchart.

Out of the 152 patients, 148 are randomized and completed the trial. On an intention-to-treat (ITT) principle, 78 patients are assigned to the 4% sodium citrate group and 70 patients to the heparin group (Fig 1).

Baseline characteristics between the two groups are similar except for the catheter insertion location and APTT before locking (Table 1).

**Table 1:** Baseline characteristics of patients.

Characteristic	Heparin group	Sodium Citrate group
	(n = 70)	(n = 78)
Age, years, median (Q1,Q3) ^a^	64 (50,74)	66 (55,74)
Male, n(%)^b^	38 (54.3)	46 (59)
Length of ICU stay, days, median (Q1,Q3) ^a^	7 (7, 10)	7 (7, 9)
Main reason for ICU admission, n (%)^c^		
Intracerebral hemorrhage	14 (20)	26 (33.3)
Sepsis or septic shock	6 (8.6)	3 (3.8)
Multiple injuries	10 (14.3)	14 (17.9)
Digestive tract perforation	6 (8.6)	6 (7.7)
Bile duct stones	7 (10)	8 (10.3)
Chronic obstructive pulmonary disease	9 (12.9)	7 (9)
Other reasons	18 (25.7)	14 (17.9)
Continuous renal replacement therapy, n(%)^c^	3 (4.3)	1 (1.3)
Enoxaparin, n(%)^b^	8 (11.4)	8 (10.3)
Multidrug-resistant infection, n(%)^c^	3 (4.3)	4 (5.1)
Right side of catheter insertion direction, n(%)^c^	69 (98.6)	77 (98.7)
Catheter insertion location, n(%)^c^		
Internal jugular vein	26 (37.1)	12 (15.4)
Subclavian vein	44 (62.9)	65 (83.3)
Femoral vein	0 (0)	1 (1.3)
APTT before locking, seconds, mean ±SD ^d^	27.8±5.4	30.5±5.9
PT before locking, seconds, mean ±SD ^d^	13.4±2.2	14.2±3.3
INR before locking, mean ±SD ^d^	1.2±0.4	1.2±0.4
FIB before locking, g/L, mean ±SD ^d^	3.8±2.0	4.0±2.0
TT before locking, seconds, mean ±SD ^d^	17.1±3.0	16.8±3.7

^a^Wilcoxon Mann-Whitney test

^b^Chi-square test

^c^Fisher’s exact test

^d^Two independent samples t test

*APTT* activated partial thromboplastin time, *PT* prothrombin time, *INR* international normalized ratio, *FIB* fibrinogen, *TT* thrombin time

### 3.2. Primary endpoint

**Fig 2:**
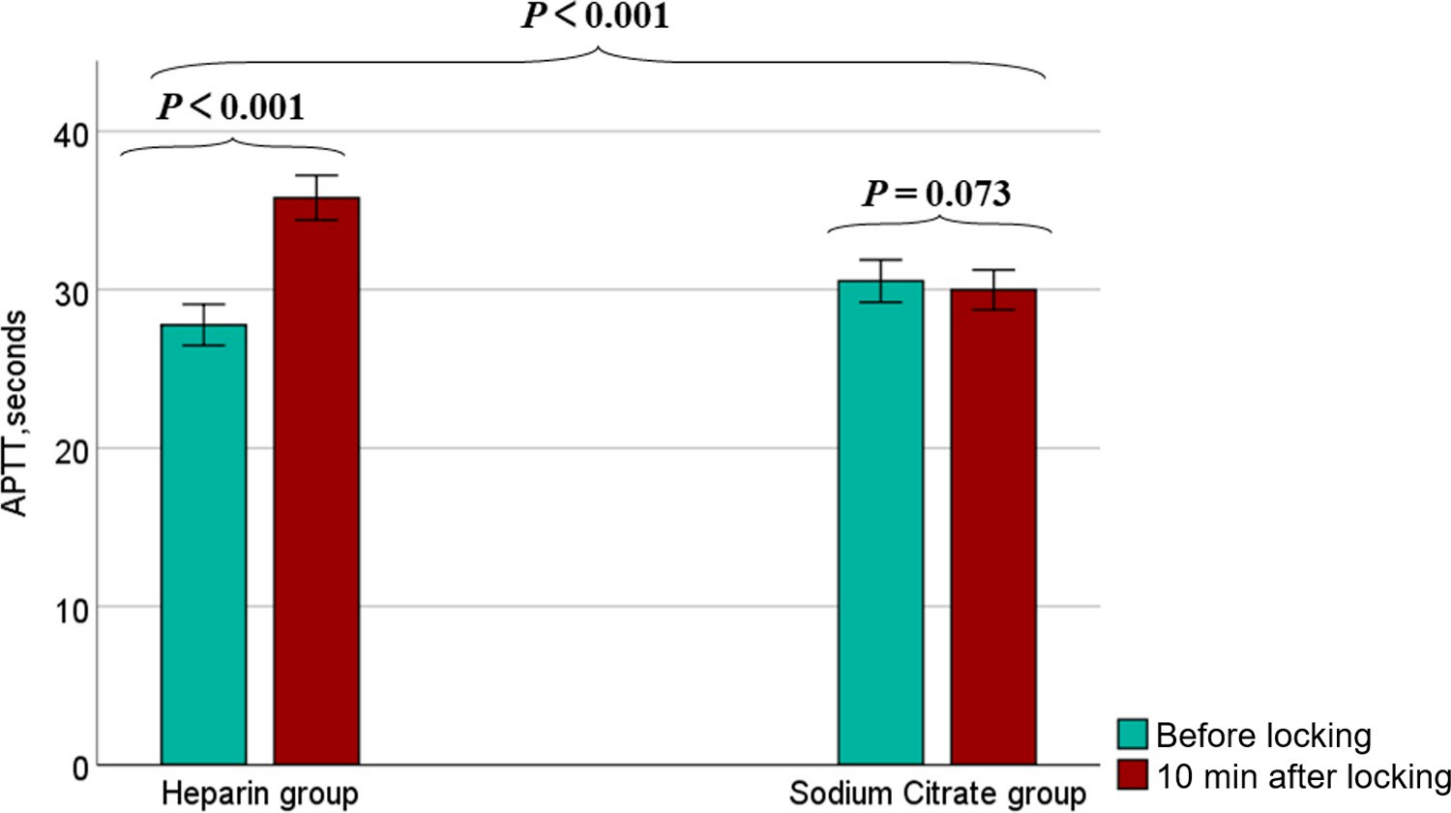
APTT 10min after locking. After adjusting for the APTT index prior to tube locking, the APTT levels were found to increase significantly in the heparin group compared to the sodium citrate group at 10 minutes after locking (LSMD = 8.15, 95%CI 7.1 to 9.2, *P*<0.001). The heparin group exhibited a significant decrease in APTT levels compared to those observed after tube locking (Difference = -8.03, 95%CI -8.93 to -7.13, *P* < 0.001). Conversely, no statistically significant difference in APTT levels was observed in the sodium citrate group (Difference = 0.56, 95%CI -0.05 to 1.17, *P* = 0.073).

After adjusting for the APTT index prior to tube locking, the APTT levels were found to increase significantly in the heparin group compared to the sodium citrate group at 10 minutes after locking (LSMD = 8.15, 95%CI 7.1 to 9.2, *P*<0.001). The heparin group exhibited a significant decrease in APTT levels compared to those observed after tube locking (Difference = -8.03, 95% CI -8.93 to -7.13, *P* < 0.001). Conversely, no statistically significant difference in APTT levels was observed in the sodium citrate group (*P* = 0.073) (Fig 2 and S1 Table).

**Fig 3:**
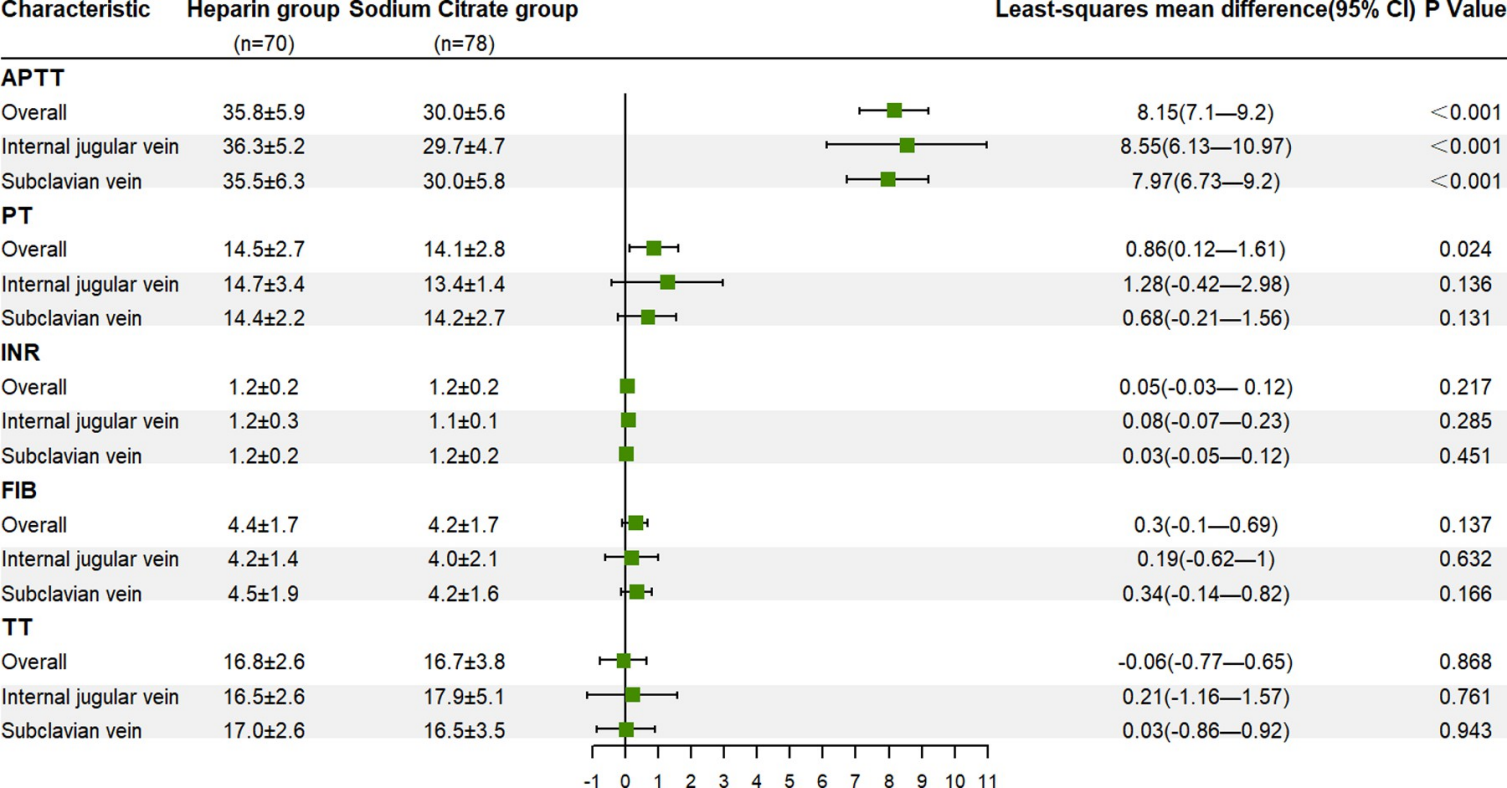
Forest map of four indexes of blood coagulation 10 minutes after locking.

Due to the uneven distribution of catheter insertion location, a post hoc analysis is performed using the internal jugular vein and subclavian vein as subgroups. In the internal jugular vein group, there was a significant increase in APTT levels in the heparin group compared to the sodium citrate group at 10 minutes after tube locking (LSMD = 8.55, 95%CI 6.13 to 10.97, *P* < 0.001). Similarly, in the subclavian vein group, the heparin group demonstrated a significant increase in APTT levels compared to the sodium citrate group at 10 minutes after locking (LSMD = 7.97, 95%CI 6.73 to 9.2, *P* < 0.001) (Fig 3). The samples of femoral vein group are too little to be included in the analysis.

### 3.3. Secondary endpoints

Among the four indexes of blood coagulation at 10 min after tube locking, after adjusting the PT index before tube locking, the heparin group increases compared with the sodium citrate group (LSMD = 0.86, 95%CI 0.12 to 1.61, *P* = 0.024) (Fig 3 and S1 Table).

Since the four indexes of blood coagulation are missing 7 d after the tube locking, the MICE method is used to fill in them. Compared with the sodium citrate group, the APTT (LSMD = 8.05, 95%CI 6.71 to 9.4, *P* < 0.001), PT (LSMD = 0.78, 95%CI 0.14 to 1.42, *P* = 0.017), and FIB (LSMD = 1.15, 95%CI 0.23 to 2.08, *P* = 0.014) of the heparin group increases (Table 2). To evaluate the robustness of this result, a sensitivity analysis is also performed (S2 Table).

**Table 2:** Four indexes of blood coagulation 7 d after the first locking.

Characteristic	Heparin group	Sodium Citrate group	*P*	Least-squares mean difference and 95%CI
	(n = 70)	(n = 78)		
APTT,seconds	35.6	29.2	<0.001	8.05(6.71–9.4)
PT,seconds	13.9	13.6	0.017	0.78(0.14–1.42)
INR	1.2	1.1	0.055	0.08(-0.002–0.15)
FIB,g/L	5.5	4.4	0.014	1.15(0.23–2.08)
TT,seconds	16.4	16.1	0.736	0.16(-0.76–1.07)

Through the implementation of analysis of covariance to adjust for the four indexes of blood coagulation prior to locking, the least-squares mean difference was employed to describe the observed outcomes. Furthermore, multivariate imputation using chained equations (MICE) methodology was applied to address missing data and ensure comprehensive data completion.

APTT activated partial thromboplastin time, PT prothrombin time, INR international normalized ratio, FIB fibrinogen, TT thrombin time

95%CI, 95% confidence interval

There are 627 catheter days in the heparin group and 674 catheter days in the sodium citrate group. The median catheter indwelling time between the two groups is 7 d, and the difference is not statistically significant (*P* = 0.456); There are 15 cases (21.4%) of catheter occlusion in the heparin group and 6 cases (7.7%) in the sodium citrate group, and the difference is statistically significant (RR = 0.36, 95%CI 0.15 to 0.87, *P* = 0.024); there is no CRBSI occurred in any of the catheters (Table 3).

**Table 3:** Other secondary endpoints and safety evaluation index.

Characteristic	Heparin group	Sodium Citrate group	*P*	Relative risk and 95%CI
	(n = 70)	(n = 78)		
Catheter indwelling time, days, median (Q1,Q3)	7 (7, 9.25)	7 (7, 9)	0.456^a^	
Catheter occlusion rate, n(%)	15 (21.4)	6 (7.7)	0.024^b^	0.36 (0.15–0.87)
CRBSI rate, n(%)	0	0		
Bleeding around the puncture site and subcutaneous hematoma rate, n(%)	9 (12.9)	1 (1.3)	0.027^b^	0.1 (0.01–0.77)
Gastrointestinal bleeding rate, n(%)	1 (1.4)	0	0.473	
Rate of ionized calcium < 1.0 mmol/L, n(%)	12 (17.1)	9 (11.5)	0.333^b^	0.67 (0.3–1.5)

^a^Wilcoxon Mann-Whitney test

^b^The relative risk and its 95%CI were calculated using modified Poisson regression analysis to assess the effect size.

95%CI, 95% confidence interval

### 3.4. Safety evaluation index

There are 9 cases (12.9%) of bleeding around the puncture site and subcutaneous hematoma in the heparin group, and 1 case (1.3%) in the sodium citrate group, and the difference is statistically significant (RR = 0.1, 95%CI 0.01 to 0.77, *P* = 0.027); one case of gastrointestinal bleeding occurred in the heparin group; ionized calcium <1.0 mmol/L occurred in 12 cases (17.1%) in the heparin group and 9 cases (11.5%) in the sodium citrate group, and the difference is not statistically significant (RR = 0.67, 95%CI 0.3 to 1.5, *P* = 0.333) (Table 3).

## 4. Discussion

A pioneering pragmatic randomized controlled trial has been designed to compare the effects of 10 U/mL heparin saline and 4% sodium citrate as locking solutions for CVCs (excluding dialysis catheters).

After using heparin to lock the tube, it will affect the four indexes of blood coagulation, resulting in elevated APTT (LSMD = 0.86, 95%CI 0.12 to 1.61, *P* = 0.024), PT (LSMD = 0.86, 95%CI 0.12 to 1.61, *P* = 0.024), while sodium citrate does not. The bleeding around the puncture site and subcutaneous hematoma rate in the sodium citrate group is also lower than that in the heparin group (RR = 0.1, 95%CI 0.01 to 0.77, *P* = 0.027). One case of gastrointestinal bleeding occurred in the heparin group. The above results show that the use of heparin to lock the tube increase the risk of bleeding, while the use of 4% sodium citrate to lock the tube did not. The similar results are also found in the dialysis catheters. Sheng demonstrated that sodium citrate significantly reduces bleeding events compared with heparin (RR = 0.36, 95%CI 0.22 to 0.60) [37], and a Meta-analysis of 13 randomized controlled trials (1770 patients) also confirmed this result (RR = 0.48, 95%CI 0.30 to 0.76) [32]. The difference of effects is closely related to the anticoagulation mechanism of both drugs. Heparin is a polysaccharide that exerts its anticoagulant effect by accelerating the activity of antithrombin III to inactivate thrombin [8]. This conformational change in antithrombin III accelerates its ability to inactivate thrombin (factor IIa), factor IXa, and factor Xa [47]. Systemic anticoagulation can be induced when locking fluid escapes from the catheter. Calcium plays a key role in thrombosis through the exogenous pathway by mediating the binding between tissue factor and factor VII, and sodium citrate as a chelator can combine with ionized calcium, thereby inhibiting blood coagulation and producing an anticoagulant effect [29]. When this chelate escapes from the catheter tip into the bloodstream, it is rapidly metabolized by the liver to sodium bicarbonate, thereby losing its anticoagulant properties and reducing the risk of bleeding [29]. In the context of CVCs (excluding dialysis catheters), it has been suggested by scholars that the advantages of sodium citrate may predominantly lie in their impact on biofilm and bacterial activity, rather than their anticoagulant properties [38]. However, our study substantiates the ongoing efficacy of sodium citrate in reducing the risk of bleeding associated with CVCs (excluding dialysis catheters). This finding necessitates further investigation and scholarly discourse to elucidate underlying mechanisms.

In patients infused with CVCs, catheter occlusion rate using heparin-locking solutions has been estimated in the range of 0% to 33% [2]. In our study, the catheter occlusion rate is 21.4% in the heparin group, and the catheter occlusion rate is reduced with sodium citrate (7.7%) (RR = 0.36, 95%CI 0.15 to 0.87, *P* = 0.024). This is because sodium citrate can inhibit the formation of biofilm on the catheter material, but heparin has an enhanced effect, and the biofilm fragments can cause occlusion of the catheter when they fall off [20, 29]. Heparin is interfered with by antithrombin III levels, and the low antithrombin III levels commonly seen in ICU patients limit the anticoagulant effect of heparin [48]. Our result is inconsistent with Wang’s findings (RR = 1.14, 95%CI 0.76 to 1.69) [8]. In dialysis catheters, the concentration of heparin locking solution is 1000–10000 U/mL [8]. However, for ICU patients infused with CVCs, the concentration of the heparin-locking solution is 10 U/mL [11]. This difference may stem from the patient’s conditions, the structure of the catheter, or the concentration of the heparin locking solution.

Theoretically, the use of sodium citrate as a tube locking solution can reduce the CRBSI rate because metal ions play a key role in stabilizing the extracellular matrix of microbial biofilms, in which sodium citrate exhibits antibacterial activity by depriving microorganisms of these essential metal ions without worrying about bacterial resistance [49]. At the concentrations > 0.5%, sodium citrate has been shown to inhibit the formation of staphylococcal biofilms [49], in which staphylococcal are responsible for approximately 35% of CRBSI [21]. Contamination during the preparation of heparin solution is also likely to cause CRBSI. In dialysis catheters, sodium citrate locking solutions have shown antibacterial properties [27, 30–33, 37]. A review of 27 studies (3003 participants) showed that sodium citrate is associated with a reduction in CRBSI compared with heparin (RR 0.49, 95%CI 0.36 to 0.68) in dialysis catheters [8]. However, no CRBSI occurs in this study, which may be related to our series based on practice-based interventions to reduce CRBSI [6]. A professional team is established to insert and care for CVCs, and the personnel has been properly educated and trained. All CVCs are inserted using maximum sterile barrier precautions, including sterile gloves, long-sleeved sterile gowns, caps, masks, and long sterile therapeutic drapes that cover the patient from head to toe, and a central venipuncture bag and bedside checklist are used. Subclavian vein catheterization is used in most of the patients (73.6%). The need for CVCs is assessed every day, and the CVCs are removed as soon as possible when not in use. In our patients, the indwelling time of CVCs for less than 7 d accounted for 63.5%. Strictly practice of hand hygiene is conducted, central venous dressing kits for catheter maintenance are used, and the integrity of catheter dressings is maintained. It is more possible that the antibacterial activity of sodium citrate can be shown more in the high-incidence area of CRBSI.

Regarding safety, because most of our patients are in a state of sedation and analgesia or coma, it is impossible to ask the patients about their perioral numbness. Fortunately, ionized calcium is monitored, finding that the rate of ionized calcium < 1.0 mmol/L in the heparin group and the sodium citrate group is similar (*P* = 0.333, RR = 0.67, 95%CI 0.3 to 1.5). At present, the reports of adverse events of citrate tube locking solution are only found in the study of high concentration (46.7%) solution, which may be related to the transient rapid decrease of ionized calcium and ionized magnesium in patients [29, 50]. An in-vitro study [51] showed that the mean ionized calcium concentrations measured at the catheter tip are 0.457 and 0.058 mmol/L when 4% and 30% sodium citrate are used, respectively (*P* < 0.001). It shows that the use of 4% sodium citrate to lock the tube is safe in clinical practice. However, heparin has been associated with many adverse events, including major bleeding, heparin-induced thrombocytopenia, thrombosis, and osteoporosis [8]. A 61-year-old male patient is recently reported in Japan who developed anaphylactic shock with heparin saline (50 units/5 mL) flushing CVCs [52], increasing the risk of heparin use.

We have to acknowledge some limitations to our study. The study is conducted in a tertiary hospital with a dedicated team of physicians and an intravenous access support team, factors that may have positively affected the results. Due to the simple randomization method used in our study, the two baseline data, APTT and catheter insertion location, are inevitably distributed unevenly, so we used some statistical methods to adjust these confounding factors, such as ex ante analysis of covariance and post hoc subgroup analysis. We had 21 patients (14.2%) who withdrew from the study within the 7-day observation period (transferred from the ICU, died, or had the catheter pulled out), so the data for the four indexes of blood coagulation 7 d after the locking of the catheter contained missing values. The missing data are 9 cases (12.9%) in the heparin group and 12 cases (15.4%) in the sodium citrate group, respectively. We filled in the missing data and performed a sensitivity analysis to evaluate the robustness of the results. The sample size calculation is only performed for main outcome indicator, and the absence of sample size calculation for other outcome measures may have compromised statistical power and increased the risk of type II error, potentially resulting in false-negative findings. There are also some regrets that we did not collect whether blood products and parenteral nutrition are infused, which are risk factors for catheter occlusion. A data safety monitoring board is not used in this study since no interim analyses were undertaken.

## 5. Conclusions

In this prospective, triple-blind, randomized, parallel-group, standard-controlled, single-center trial, the efficacy and safety of 4% sodium citrate are demonstrated as a tube locking solution. In ICU patients receiving CVCs infusion, the use of 4% sodium citrate to lock the catheter can reduce the risk of bleeding and catheter occlusion without the occurrence of hypocalcemia.

Our work show that the 4% sodium citrate is possible to replace heparin as a tube-locking solution for CVCs (excluding dialysis catheters). Hence, we propose that additional prospective studies be done, which (i) use the same primary outcome, (ii) allow broader inclusion criteria, and (iii) involve multiple centres.

## Supporting information

S1 Table Four indexes of blood coagulation 10 minutes after locking. (DOCX)

S2 Table Four indexes of blood coagulation 7 days after the first locking. (DOCX)

S1 File CONSORT 2010 checklist of information to include when reporting a randomised trial. (DOC)

S2 File Team composition and overall research procedures. (DOCX)

S3 File Flushing and locking procedures. (DOCX)

S4 File (DOCX)

S5 File (DOCX)

## Abbreviations

CVCs: Central venous catheters
ICU: Intensive care unit
CRBSI: Catheter-related bloodstream infection
HIT: Heparin-induced thrombocytopenia
APTT: Activated partial thromboplastin time
PT: Prothrombin time
INR: International normalized ratio
FIB: Fibrinogen
TT: Thrombin time
RR: Relative risk
LSMD: Least-squares mean difference
