# Supplementary material for: Clinical application of 4% sodium citrate and heparin in the locking of central venous catheters (excluding dialysis catheters) in intensive care unit patients: A pragmatic randomized controlled trial
Source: PLoS One. 2023 Jul 3;18(7):e0288117. doi: 10.1371/journal.pone.0288117 (PMC10317237; doi:10.1371/journal.pone.0288117)
Supplement: S1 Table — (DOCX) [file pone.0288117.s001.docx]

**S1 Table** Four indexes of blood coagulation 10 minutes after locking.

| Characteristic | Heparin group | Sodium Citrate group | *P* | Least-squares mean difference and 95% CI |
| --- | --- | --- | --- | --- |
|  | (n=70) | (n=78) |  |  |
| APTT before locking,seconds | 27.8±5.4 | 30.5±5.9 | 0.004^c^ |  |
| APTT 10min after locking,seconds | 35.8±5.9 | 30.0±5.6 | ＜0.001^a^ | 8.15 (7.1 — 9.2) |
| *P* | ＜0.001^b^ | 0.073^b^ |  |  |
| difference and 95% CI | -8.03 (-8.93 — -7.13) | 0.56 (-0.05 —1.17) |  |  |
| PT before locking,seconds | 13.4±2.2 | 14.2±3.3 | 0.075^c^ |  |
| PT 10min after locking,seconds | 14.5±2.7 | 14.1±2.8 | 0.024^a^ | 0.86 (0.12 — 1.61) |
| *P* | ＜0.001^b^ | 0.749^b^ |  |  |
| difference and 95% CI | -1.14 (-1.75 — -0.53) | 0.1 (-0.5 — 0.69) |  |  |
| INR before locking | 1.2±0.4 | 1.2±0.4 | 0.401^c^ |  |
| INR 10min after locking | 1.2±0.2 | 1.2±0.2 | 0.217^a^ | 0.05（-0.03 — 0.12） |
| *P* | 0.204^b^ | 0.521^b^ |  |  |
| difference and 95% CI | -0.06 (-0.16 — 0.04) | 0.03 (-0.06 — 0.12) |  |  |
| FIB before locking,g/L | 3.8±2.0 | 4.0±2.0 | 0.595^c^ |  |
| FIB 10min after locking,g/L | 4.4±1.7 | 4.2±1.7 | 0.137^a^ | 0.3（-0.1— 0.69） |
| *P* | 0.002^b^ | 0.123^b^ |  |  |
| difference and 95% CI | -0.6 (-0.96 — -0.24) | -0.23 (-0.53 — 0.06) |  |  |
| TT before locking,seconds | 17.1±3.0 | 16.8±3.7 | 0.638^c^ |  |
| TT 10min after locking,seconds | 16.8±2.6 | 16.7±3.8 | 0.868^a^ | -0.06 (-0.77— 0.65) |
| *P* | 0.349^b^ | 0.581^b^ |  |  |
| difference and 95% CI | 0.27 (-0.31 — 0.85) | 0.14 (-0.37 — 0.66) |  |  |

^a^We adjusted the four indexes of blood coagulation before locking by analysis of covariance

^b^Paired t test

^c^Two independent samples t test

*APTT* activated partial thromboplastin time,*PT* prothrombin time,*INR* international normalized ratio,*FIB* fibrinogen ,*TT* thrombin time

*95%CI* 95% confidence interval
