# Supplementary material for: Clinical application of 4% sodium citrate and heparin in the locking of central venous catheters (excluding dialysis catheters) in intensive care unit patients: A pragmatic randomized controlled trial
Source: PLoS One. 2023 Jul 3;18(7):e0288117. doi: 10.1371/journal.pone.0288117 (PMC10317237; doi:10.1371/journal.pone.0288117)
Supplement: S2 Table — (DOCX) [file pone.0288117.s002.docx]

**Sensitivity analysis**

We used the multivariate imputation by chained equations to impute the missing values of the four indexes of blood coagulation 7 days after the tube locking. Compared with the sodium citrate group, the APTT (LSMD = 8.05, 95%CI 6.71 to 9.4, *P* < 0.001), PT (LSMD = 0.78, 95%CI 0.14 to 1.42, *P* = 0.017), FIB (LSMD = 1.15, 95%CI 0.23 to 2.08, *P* = 0.014) all increased, which are similar to the results before filling, demonstrating the robustness of the results.

**S2 Table** Four indexes of blood coagulation 7 days after the first locking.

| Characteristic |  | Heparin group | Sodium Citrate group | *P* | Least-squares mean difference and 95% CI |
| --- | --- | --- | --- | --- | --- |
|  |  | (n=70) | (n=78) |  |  |
| APTT,seconds | After data filling | 35.6 | 29.2 | ＜0.001 | 8.05（6.71—9.4） |
|  | Before data filling | 36.2±5.1 | 28.6±5.3 | ＜0.001 | 10.32（9.23—11.41） |
| PT,seconds | After data filling | 13.9 | 13.6 | 0.017 | 0.78（0.14—1.42） |
|  | Before data filling | 13.9±2.3 | 13.5±2.9 | 0.006 | 0.93（0.28—1.59） |
| INR | After data filling | 1.2 | 1.1 | 0.055 | 0.08（-0.002—0.15） |
|  | Before data filling | 1.2±0.2 | 1.1±0.2 | ＜0.001 | 0.11（0.05—0.17） |
| FIB,g/L | After data filling | 5.5 | 4.4 | 0.014 | 1.15（0.23—2.08） |
|  | Before data filling | 5.6±3.8 | 4.3±1.8 | 0.007 | 1.43（0.4—2.45） |
| TT,seconds | After data filling | 16.4 | 16.1 | 0.736 | 0.16（-0.76—1.07） |
|  | Before data filling | 16.4±3.0 | 16.1±3.5 | 0.816 | 0.12（-0.9—— 1.14） |

We adjusted the four indexes of blood coagulation before locking by analysis of covariance, then using multivariate imputation by chained equations to fill in data

*APTT* activated partial thromboplastin time, *PT* prothrombin time, *INR* international normalized ratio, *FIB* fibrinogen, *TT* thrombin time

95%CI, 95% confidence interval
