# Supplementary material for: Clinical application of 4% sodium citrate and heparin in the locking of central venous catheters (excluding dialysis catheters) in intensive care unit patients: A pragmatic randomized controlled trial
Source: PLoS One. 2023 Jul 3;18(7):e0288117. doi: 10.1371/journal.pone.0288117 (PMC10317237; doi:10.1371/journal.pone.0288117)
Supplement: S2 File — (DOCX) [file pone.0288117.s004.docx]

**Team composition and overall research procedures**

Yuchun Deng: Designing research plans, statistical data, writing papers

Ying Liu, Xiaorong Yang, Liqin Zhang: Collect, organize, and input data

Wangen Yue: Generating random schemes

Dan Liu: Managing random programs

Tingting Yao, Shimei Deng: Marking the test number and preparing the locking solution

1. Design and register the research protocol (Yuchun Deng).

2. Centralized training was given to general nurses in the intensive care department of Zhongjiang County People's Hospital who participated in this survey. The training content included the correct way of flushing and locking tubes, the pharmacological effects and adverse reactions of heparin and sodium citrate, and the maintenance of CVCs. (Yuchun Deng).

3. Generating a random plan, and the grouping is replaced by 1 and 2 (Wangen Yue).

4. Managing the random program (Dan Liu), and unblinding the peoples who marked the test number and prepared the tube locking solution (Tingting Yao, Shimei Deng).

5. The prepared tube locking solution is handed over to the nurses managing hospital beds for locking and tube locking operations.

6. Two of the investigators (Ying Liu, Xiaorong Yang, Liqin Zhang) used the patient medical data sheet to evaluate the patient's CVCs and record the observation results every day. And the data is sorted and entered, and the grouping is replaced by 1 and 2.

7. Performing the statistical analysis (Yuchun Deng).

8. After the statistical results came out, those who managed the random program (Dan Liu) were unblinded. Group 1 was given 10u/mL heparin tube locking solution, and group 2 was given 4% sodium citrate tube locking solution.
