# Supplementary material for: Clinical application of 4% sodium citrate and heparin in the locking of central venous catheters (excluding dialysis catheters) in intensive care unit patients: A pragmatic randomized controlled trial
Source: PLoS One. 2023 Jul 3;18(7):e0288117. doi: 10.1371/journal.pone.0288117 (PMC10317237; doi:10.1371/journal.pone.0288117)
Supplement: S3 File — (DOCX) [file pone.0288117.s005.docx]

**Flushing and locking procedures**

Catheters for 24-hour drug infusion: CVCs flushing must be performed using turbulent flush with 10 mL of 0.9% sodium chloride injection when nursing shifts, with 8-hour intervals.

Intermittent use of catheters: After withdrawing the locking fluid, 10mL 0.9% sodium chloride injection was used to turbulently flush CVCs, followed by infusion, IV push, parenteral nutrition, transfusion of blood products. Subsequently, 10mL 0.9% sodium chloride injection was still used to turbulently flush CVCs, then the experimental group was treated with sodium citrate locking solution, and the control group was treated with heparin locking solution, in which the positive-pressure locking techniques were used.

Unused catheters: After withdrawing the locking fluid, 10 mL of 0.9% sodium chloride injection was used to turbulently flush CVCs, then the experimental group was treated with sodium citrate locking solution, the control group was treated with heparin locking solution, in which the positive-pressure locking techniques was used, and the locking with an interval of 24 hours.
