# Supplementary material for: Clinical application of 4% sodium citrate and heparin in the locking of central venous catheters (excluding dialysis catheters) in intensive care unit patients: A pragmatic randomized controlled trial
Source: PLoS One. 2023 Jul 3;18(7):e0288117. doi: 10.1371/journal.pone.0288117 (PMC10317237; doi:10.1371/journal.pone.0288117)
Supplement: S4 File — (DOCX) [file pone.0288117.s006.docx]

1. Main objectives

To evaluate the feasibility of replacing heparin saline with 4% sodium citrate injection as the locking fluid for central venous catheters (not for hemodialysis).

1. Research content

To evaluate whether sodium citrate lock solution can reduce the risk of bleeding, catheter blockage, and the incidence of catheter-related bloodstream infection compared with heparin lock solution.

1. Key technology

1. Research methods. A prospective, triple-blind, randomized, parallel-group, standard-controlled, single-center clinical study conducted in accordance with the reporting specification for randomized controlled trials (CONSORT statement).

2. Research object. It is planned to select patients with indwelling CVC catheter for infusion in Department of Critical Care Medicine, People's Hospital of Zhongjiang County from December 2021 to July 2022 as the research subjects.

① Inclusion criteria: Patients aged 18-80 years old who used a CVC for infusion during their stay in the intensive care unit.

② Exclusion criteria: Pregnant, perinatal and lactating women, those who are allergic to heparin or sodium citrate, or those with abnormal coagulation function.

3. Intervention measures. According to the ratio of 1:1, the patients were divided into the experimental group and the control group by simple random method.

① Test group: 5 mL of 200 mL of 4% sodium citrate injection was used as a lock solution.

② Control group: Take 0.4mL of 12500u/2mL heparin sodium injection, then add 0.9% sodium chloride injection 250mL, and take 5mL after the preparation is uniform. The heparin concentration in the locking solution is 10u/mL.

Before and after each infusion, medication, parenteral nutrition, transfusion of blood products, and replacement of pipeline equipment, 10 mL of 0.9% sodium chloride injection was used to flush the central venous catheter in a pulse-type manner. The sodium citrate lock solution was used in test group, and the heparin lock solution was used in control group, both groups used positive pressure lock. CVCs not in use were flushed and locked once a day.

4. Outcome indicators

Before locking the tube, 10 minutes after the tube was locked, and 7 days after the first tube locking, blood was drawn from the patients respectively, and four indicators of blood coagulation were compared, including activated partial thromboplastin time (APTT), thrombin time (TT), prothrombin time (PT), international normalized ratio (INR), fibrinogen quantification (FIB).

During the hospitalization in the intensive care unit, the investigator who collected the data used the patient medical data sheet to evaluate the patient's central venous catheter daily, and collected follow data: catheter indwelling time, catheter blockage rate, catheter-related blood infection rate, bleeding around the puncture site and subcutaneous hematoma rate, gastrointestinal bleeding rate, rate of ionized calcium < 1.0 mmol/L.

5. Estimation of sample size

This study is a randomized controlled trial. The test group uses 4% sodium citrate lock solution, and the control group uses 10u/mL heparin saline lock solution. The main outcome index is activated partial thromboplastin time (APTT) 10 minutes after lock. According to literature review and pre-experimental results, the mean and standard deviation of the APTT of the two groups were obtained, and the two-sided α=0.05, with a power of 90%, was calculated using the PASS 15 software to obtain the sample size of the two groups.

6. Statistical methods

The collected data were double-entry, and based on the intention-to-treat principle (ITT), data analysis was carried out using SPSS 25.0 and R language statistical software (R, v.4.1.2). Continuity variables are tested by histogram and Shapiro-Wilk test, and those that conformed to normal distribution are expressed as mean and standard deviation (SD); those that did not conform to normal distribution are expressed as median and quartile spacing. When comparing continuity variables, the least mean difference (LMD) and its 95% confidence interval (95% Cl) are calculated by analysis of covariance if it is conformed to the normal distribution. Missing values are imputed using the multivariate imputation by chained equations (MICE) method under the assumption of missing at random, and a sensitivity analysis is performed to evaluate the robustness of the results. The Wilcoxon Mann-Whitney test is used for those that do not conform to the normal distribution. Categorical data are presented in frequencies and percentages, and the Chi-square test or Fisher's exact test is used. The relative risk (RR) and its 95% confidence interval (95% Cl) are calculated using modified Poisson regression. All statistical analyses are based on two-sided hypothesis testing, with α = 0.05 as the test level, and *P* ≤ 0.05 as statistically significant.

1. Research Background

1. Necessity of project approval

A central venous catheter (CVC) is a catheter inserted through the subclavian, internal jugular, or femoral veins with the tip in the superior or inferior vena cava [1]. CVCs are a common method in modern critical care medicine to treat critically ill patients, and are widely used in: ①hemodynamic monitoring; ②blood purification; ③injection of blood products, drugs, and total parenteral nutrition. Once catheter-related bloodstream infection [2, 3], catheter blockage, and bleeding complications occur, it will not only affect the treatment effect of the primary disease, prolong the length of hospital stay, increase the mortality rate of patients, but also cause waste of medical resources and increase hospitalization costs [4-6]. Locking technology is an important link to ensure the patency of catheters, effectively prevent thrombosis, prevent bleeding complications and catheter-related bloodstream infections, and enable the continuous and effective use of CVCs.

Currently, heparin saline is used clinically as the lock solution for central venous catheters (not for hemodialysis) [7, 8]. However, heparin has many disadvantages as a lock solution [4, 9]: ①It causes systemic anticoagulation, especially in the intensive care unit, some high-risk patients (severe sepsis patients, postoperative patients, etc.) will increase the risk of bleeding [10]; ② causing heparin-induced thrombocytopenia [11, 12]; ③ promoting S. aureus biofilm formation in a dose-dependent manner [13] and increases catheter-associated bloodstream infection and catheter blockage [14-16]. To improve above conditions, researchers have been searching for a safe alternative to heparin. Sodium citrate lock solution has local anticoagulant properties, has no effect on the coagulation function of the body [17-19], and can reduce the risk of bleeding. As a high antibacterial concentration lock solution, it can prevent the formation of microbial biofilm, inhibit the growth of staphylococcus aureus and staphylococcus epidermidis, thereby reducing catheter blockage and catheter-related bloodstream infection, and there is no need to worry about the emergence of bacterial resistance [15, 20, 21]. In international guidelines, sodium citrate at a concentration of 4% has the best cost-effectiveness and safety [16].

In order to evaluate the efficacy and safety of 4% sodium citrate lock solution in central venous catheters (not for hemodialysis), it is necessary to conduct this study.

2. Research status at home and abroad

At present, many studies have compared the effect of using sodium citrate lock solution and heparin lock solution in CVC lock in hemodialysis patients, suggesting that sodium citrate lock is not only effective, but also safer [6, 9, 18 , 20, 22]. The study by Kaixiang Sheng [23] proved that the use of sodium citrate to lock the catheter can reduce the risk of bleeding (RR=0.36, 95% Cl 0.22-0.60), and a META analysis including 13 randomized controlled trials (1770 patients) also confirmed the result (RR=0.48, 95%CI 0.30-0.76) [20]. The research results of Ying Wang [9] showed that the rate of catheter blockage was consistent between sodium citrate lock and heparin lock (RR=1.14, 95%Cl 0.76-1.69). A review of 27 studies (3003 participants) showed that sodium citrate reduced catheter-associated bloodstream infection compared with heparin (RR=0.49, 95% CI 0.36-0.68) [9].

The use of sodium citrate lock solution for non-hemodialysis central venous catheters has also been advocated by experts [30], but there are no clinical studies in this area.

3. Development trend

There have been more and more evidences that heparin has many disadvantages as a lock solution for CVCs, and finding a safer and more effective lock solution is needed. In order to reduce the risk of bleeding and heparin-induced thrombocytopenia, some scholars proposed to use normal saline instead of heparin as the lock fluid for central venous catheters[24-26]. However, normal saline has no anticoagulant effect and may increase the blockage rate of CVCs. A meta-analysis of 10 studies with 1672 participants showed that the blockage rate of heparin was lower than that of normal saline (RR=0.7, 95% Cl 0.51-0.95, P=0.02) [27]. Some scholars have also proposed the use of antibiotic lock solution to reduce catheter-related bloodstream infection, but this approach may promote the emergence of drug-resistant bacteria [28, 29].

With its antibacterial and local anticoagulant properties, sodium citrate is expected to replace heparin as a new generation of ideal lock solution.

References：

[1]. Group of experts on Safety Management of Central Venous access Devices. The expert consensus on safety management of central venous access device (2019 edition). Chinese Journal of Surgery. 2020. 58(4): p. 261-272.

[2]. Timsit, J.F., et al., Bloodstream infections in critically ill patients: an expert statement. Intensive Care Med, 2020. 46(2): p. 266-284.

[3]. Rupp, M.E. and R. Karnatak, Intravascular Catheter-Related Bloodstream Infections. Infect Dis Clin North Am, 2018. 32(4): p. 765-787.

[4]. Zhong, L., et al., Normal saline versus heparin for patency of central venous catheters in adult patients - a systematic review and meta-analysis. Critical care (London, England), 2017. 21(1): p. 5-5.

[5]. Rosenthal, V.D., et al., International Nosocomial Infection Control Consortium (INICC) report, data summary of 45 countries for 2012-2017: Device-associated module. Am J Infect Control, 2020. 48(4): p. 423-432.

[6]. Gudiol, C., et al., A Randomized, Double-Blind, Placebo-Controlled Trial (TAURCAT Study) of Citrate Lock Solution for Prevention of Endoluminal Central Venous Catheter Infection in Neutropenic Hematological Patients. Antimicrob Agents Chemother, 2020. 64(2).

[7]. Intravenous Therapy Committee of Chinese Nursing Association. Expert consensus on venous catheter maintenance. Chinese Journal of Nursing. 2019. 54(9): p. 1334-1342.

[8]. Gorski, L.A., A Look at 2021 Infusion Therapy Standards of Practice. Home Healthc Now, 2021. 39(2): p. 62-71.

[9]. Wang, Y., et al., Anticoagulants and antiplatelet agents for preventing central venous haemodialysis catheter malfunction in patients with end-stage kidney disease. Cochrane Database Syst Rev, 2016. 4: p. CD009631.

[10]. Bovet, J., et al., Evaluation of anti-Xa activity after injection of a heparin lock for dialysis catheters in intensive care: A prospective observational study. Thromb Res, 2020. 188: p. 82-84.

[11]. Kelton, J.G. and T.E. Warkentin, Heparin-induced thrombocytopenia: a historical perspective. Blood, 2008. 112(7): p. 2607-16.

[12]. Linkins, L.A., et al., Treatment and prevention of heparin-induced thrombocytopenia: Antithrombotic Therapy and Prevention of Thrombosis, 9th ed: American College of Chest Physicians Evidence-Based Clinical Practice Guidelines. Chest, 2012. 141(2 Suppl): p. e495S-e530S.

[13]. SHANKS, R.M.Q., et al., Heparin Stimulates Staphylococcus aureus Biofilm Formation. Infection and Immunity, 2005. 73(8): p. 4596-4606.

[14]. Moran, J.M.B., et al., A Randomized Trial Comparing Gentamicin/Citrate and Heparin Locks for Central Venous Catheters in Maintenance Hemodialysis Patients. American journal of kidney diseases, 2011. 59(1): p. 102-107.

[15]. Shanks, R.M.Q., et al., Catheter lock solutions influence staphylococcal biofilm formation on abiotic surfaces. Nephrology Dialysis Transplantation, 2006. 21(8): p. 2247-2255.

[16]. Szymańska, J., et al., Locked Away—Prophylaxis and Management of Catheter Related Thrombosis in Hemodialysis. Journal of clinical medicine, 2021. 10(11): p. 2230.

[17]. Hermite, L., et al., Sodium citrate versus saline catheter locks for non-tunneled hemodialysis central venous catheters in critically ill adults: a randomized controlled trial. Intensive Care Med, 2012. 38(2): p. 279-85.

[18]. Quenot, J.P., et al., Trisodium citrate 4% versus heparin as a catheter lock for non-tunneled hemodialysis catheters in critically ill patients: a multicenter, randomized clinical trial. Ann Intensive Care, 2019. 9(1): p. 75.

[19]. Honore, P.M., et al., What should be the best dialysis catheter lock in critically ill patients? Crit Care, 2019. 23(1): p. 339.

[20]. Zhao, Y., et al., Citrate versus heparin lock for hemodialysis catheters: a systematic review and meta-analysis of randomized controlled trials. Am J Kidney Dis, 2014. 63(3): p. 479-90.

[21]. Correa Barcellos, F., et al., Comparative effectiveness of 30 % trisodium citrate and heparin lock solution in preventing infection and dysfunction of hemodialysis catheters: a randomized controlled trial (CITRIM trial). Infection, 2016. 45(2): p. 139-145.

[22]. Pierce, D.A. and M.V. Rocco, Trisodium Citrate: An Alternative to Unfractionated Heparin for Hemodialysis Catheter Dwells. Pharmacotherapy, 2010. 30(11): p. 1150-1158.

[23]. Sheng, K.X., et al., Comparative efficacy and safety of lock solutions for the prevention of catheter-related complications including infectious and bleeding events in adult haemodialysis patients: a systematic review and network meta-analysis. Clin Microbiol Infect, 2020. 26(5): p. 545-552.

[24]. Perez-Granda, M.J., et al., Randomized clinical trial analyzing maintenance of peripheral venous catheters in an internal medicine unit: Heparin vs. saline. PLoS One, 2020. 15(1): p. e0226251.

[25]. Schallom, M.E., et al., Heparin or 0.9% sodium chloride to maintain central venous catheter patency: a randomized trial. Crit Care Med, 2012. 40(6): p. 1820-6.

[26]. Goossens, G.A., et al., Comparing normal saline versus diluted heparin to lock non-valved totally implantable venous access devices in cancer patients: a randomised, non-inferiority, open trial. Ann Oncol, 2013. 24(7): p. 1892-1899.

[27]. Lopez-Briz, E., et al., Heparin versus 0.9% sodium chloride locking for prevention of occlusion in central venous catheters in adults. Cochrane Database Syst Rev, 2018. 7: p. CD008462.

[28]. Landry, D.L., et al., Emergence of gentamicin-resistant bacteremia in hemodialysis patients receiving gentamicin lock catheter prophylaxis. Clin J Am Soc Nephrol, 2010. 5(10): p. 1799-804.

[29]. Dixon, J.J., M. Steele and A.D. Makanjuola, Anti-microbial locks increase the prevalence of Staphylococcus aureus and antibiotic-resistant Enterobacter: observational retrospective cohort study. Nephrol Dial Transplant, 2012. 27(9): p. 3575-81.

[30]. Pittiruti, M., et al., Evidence-based criteria for the choice and the clinical use of the most appropriate lock solutions for central venous catheters (excluding dialysis catheters): a GAVeCeLT consensus. J Vasc Access, 2016. 17(6): p. 453-464.
