# Supplementary material for: Clinical application of 4% sodium citrate and heparin in the locking of central venous catheters (excluding dialysis catheters) in intensive care unit patients: A pragmatic randomized controlled trial
Source: PLoS One. 2023 Jul 3;18(7):e0288117. doi: 10.1371/journal.pone.0288117 (PMC10317237; doi:10.1371/journal.pone.0288117)
Supplement: S5 File — (DOCX) [file pone.0288117.s007.docx]

1. 主要目标

评估4%枸橼酸钠注射液代替肝素盐水成为中心静脉导管(非血透用)封管液的可行性。

（二）研究内容

与肝素封管液相比，评估枸橼酸钠封管液能否降低出血风险、堵管、导管相关血流感染的发生率。

（三）技术关键

1.研究方法 按照随机对照试验报告规范（CONSORT声明）进行的前瞻性、三盲、随机、平行分组、标准对照、单中心试验的临床研究。

2.研究对象：拟选择2021年12月—2022年7月中江县人民医院重症医学科留置CVC导管进行输液的患者作为研究对象。

① 纳入标准 年龄18~80周岁，在重症监护室住院期间使用中心静脉导管进行输液的患者。

② 排除标准 妊娠期、围产期、哺乳期的妇女，对肝素或枸橼酸钠过敏者，或者凝血功能异常者。

3.干预措施 按照1：1比例使用简单随机的方法分为试验组及对照组。

① 试验组：200mL 4%枸橼酸钠注射液取5mL作为封管液。

② 对照组：12500u/2mL肝素钠注射液取0.4mL 加入0.9%氯化钠注射液250mL，配制均匀后取5mL，封管液中肝素浓度为10u/mL。

每次输液、用药、肠外营养、输入血液制品及更换管路设备前后均使用10mL 0.9%氯化钠注射液脉冲式冲洗中心静脉导管，试验组使用枸橼酸钠封管液，对照组使用肝素封管液，运用正压封管；暂未使用的中心静脉导管每日冲管、封管一次。

4.结局指标

于封管前，封管后 10分钟，首次封管后 7天，分别抽取患者的血液，比较血凝四项指标，包括活化部分凝血活酶时间(APTT)、凝血酶时间(TT)、凝血酶原时间(PT)、国际标准化比值（INR）、纤维蛋白原定量（FIB）。

在重症监护室住院期间，由收集资料的调查员使用患者医疗数据表每日对患者的中心静脉导管进行评估，收集导管留置时间、导管堵塞率、导管相关血液感染发生率、穿刺点周围渗血及皮下血肿发生率、消化道出血发生率、 离子钙＜1.0mmol/L 发生率。

5.样本量的估算

本研究为随机对照试验，试验组使用4%枸橼酸钠封管液，对照组使用10u/mL肝素盐水封管液，研究对象的封管后10分钟活化部分凝血活酶时间(APTT)为观测的主要结局指标，根据查阅文献以及预实验结果得出两组APTT的均数与标准差，设双侧α=0.05，把握度为90%，利用PASS 15软件计算得到两组的样本量。

6.统计学方法

所收集数据进行双人录入，基于意向性分析原则（ITT），运用SPSS 25.0与R语言统计学软件 (R, v.4.1.2)开展数据分析。连续性变量经直方图及 Shapiro-Wilk 检验，符合正态分布，以均数±标准差表示，不符合正态分布，以中位数（四分位数间距）表示。比较连续性变量时，符合正态分布的采用协方差分析计算效应值最小二乘均数差值及其95%置信区间，缺失值在随机缺失假设下采用了MICE法进行填补，并进行敏感性分析以评价结果的稳健性，不符合正态分布的采取秩和检验；分数变量采用例数(百分比)表示，采用卡方检验或者 Fisher 确切概率法。运用修正 Poisson 回归计算出效应值相对危险度（RR）及其95%置信区间。所有统计分析基于双侧假设检验，以 α=0.05 为 检验水准，以 *P*≤0.05 为差异有统计学意义。

1. 研究背景
2. 立项的必要性

中心静脉导管是经锁骨下静脉、颈内静脉、股静脉置入，尖端位于上腔静脉或下腔静脉的导管[1]。是现代重症医学救治急危重患者的常用方法，被广泛使用于：①血流动力学监测；②血液净化；③注射血液制品、药物以及全肠外营养等方面。一旦发生导管相关血流感染[2, 3]、导管堵塞、出血并发症等，不仅影响原发病的治疗效果、延长住院时间、增加患者病死率，还会造成医疗资源的浪费、增加住院费用[4-6]。封管技术是确保导管通畅、有效预防血栓形成、预防出血并发症及导管相关血流感染，使中心静脉导管得以持续有效应用的一个重要环节。

目前临床使用肝素盐水作为中心静脉导管（非血透用）的封管液[7, 8]。但肝素作为封管液具有诸多劣势[4, 9]：①引起全身性抗凝，特别是在重症监护室中，有一些高危的患者（严重的脓毒血症患者、术后患者等）会增加出血风险[10]；②引发肝素诱导性血小板减少症[11, 12]；③以剂量依赖性方式促进金黄色葡萄球菌生物膜形成[13]，增加导管相关血流感染与导管堵塞[14-16]。为了改善这些情况，研究人员一直在寻找一种安全的肝素替代品。

枸橼酸钠封管液具有局部抗凝特性，对机体凝血功能无影响[17-19]，可以降低出血风险。作为高抗菌浓度封管液可以通过阻止微生物生物膜的形成，抑制金黄色葡萄球菌及表皮葡萄球菌的生长,从而降低导管堵塞与导管相关血流感染，且不用担心细菌耐药性的产生[15, 20, 21]。在国际指南中，浓度为4%的枸橼酸钠具有最佳的成本效益和安全性[16]。

为了评估4%枸橼酸钠封管液在中心静脉导管（非血透用）的治疗效果和治疗安全性，我们有必要进行这项研究。

1. 国内外研究现状

目前有众多研究比较使用枸橼酸钠封管液和肝素封管液在血液透析患者中心静脉导管封管中的效果，提示枸橼酸钠封管不仅有效，而且更安全[6, 9, 18, 20, 22]。Kaixiang Sheng[23]的研究证明使用枸橼酸钠封管可以降低出血风险（RR=0.36，95% Cl 0.22-0.60)，一项包含13项随机对照试验（1770名患者）的META分析也证实了这项结果 (RR=0.48，95%CI 0.30-0.76)[20]。Ying Wang[9]的研究结果说明使用枸橼酸钠封管与肝素封管发生导管堵塞率一致（RR=1.14，95%Cl 0.76-1.69）。一项纳入27项研究（3003名参与者）的综述表明：枸橼酸钠与肝素相比可减少导管相关血流感染（RR=0.49，95% CI 0.36-0.68）[9]。

将枸橼酸钠封管液用于非血透用中心静脉导管也得到了专家的提倡[30]，然而并没有这方面的临床研究。

1. 发展趋势

已经有越来越多的证据表明肝素作为中心静脉导管的封管液有诸多劣势，我们需要找到更加安全有效的封管液。为了减少出血风险与肝素诱导性血小板减少症，有学者提出使用生理盐水替代肝素成为中心静脉导管的封管液[24-26]，但是生理盐水并没有抗凝的效果，有可能会增加中心静脉导管的堵管率。一项包含10项研究，1672名参与者的META分析显示：肝素的堵塞率比生理盐水低（RR=0.7，95%Cl 0.51-0.95，P=0.02）[27]。也有学者提出使用抗生素封管液来降低导管相关血流感染，但这种做法可能会促进耐药细菌的产生[28, 29]。

枸橼酸钠凭借其抗菌与局部抗凝的特性，有望代替肝素成为新一代理想的封管液。

参考文献：

[1]. 中心静脉血管通路装置安全管理专家共识（2019版）. 中华外科杂志, 2020. 58(4): 第261-272页.

[2]. Timsit, J.F., et al., Bloodstream infections in critically ill patients: an expert statement. Intensive Care Med, 2020. 46(2): p. 266-284.

[3]. Rupp, M.E. and R. Karnatak, Intravascular Catheter-Related Bloodstream Infections. Infect Dis Clin North Am, 2018. 32(4): p. 765-787.

[4]. Zhong, L., et al., Normal saline versus heparin for patency of central venous catheters in adult patients - a systematic review and meta-analysis. Critical care (London, England), 2017. 21(1): p. 5-5.

[5]. Rosenthal, V.D., et al., International Nosocomial Infection Control Consortium (INICC) report, data summary of 45 countries for 2012-2017: Device-associated module. Am J Infect Control, 2020. 48(4): p. 423-432.

[6]. Gudiol, C., et al., A Randomized, Double-Blind, Placebo-Controlled Trial (TAURCAT Study) of Citrate Lock Solution for Prevention of Endoluminal Central Venous Catheter Infection in Neutropenic Hematological Patients. Antimicrob Agents Chemother, 2020. 64(2).

[7]. 中华护理学会静脉输液治疗专业委员会, 临床静脉导管维护操作专家共识. 中华护理杂志, 2019. 54(9): 第1334-1342页.

[8]. Gorski, L.A., A Look at 2021 Infusion Therapy Standards of Practice. Home Healthc Now, 2021. 39(2): p. 62-71.

[9]. Wang, Y., et al., Anticoagulants and antiplatelet agents for preventing central venous haemodialysis catheter malfunction in patients with end-stage kidney disease. Cochrane Database Syst Rev, 2016. 4: p. CD009631.

[10]. Bovet, J., et al., Evaluation of anti-Xa activity after injection of a heparin lock for dialysis catheters in intensive care: A prospective observational study. Thromb Res, 2020. 188: p. 82-84.

[11]. Kelton, J.G. and T.E. Warkentin, Heparin-induced thrombocytopenia: a historical perspective. Blood, 2008. 112(7): p. 2607-16.

[12]. Linkins, L.A., et al., Treatment and prevention of heparin-induced thrombocytopenia: Antithrombotic Therapy and Prevention of Thrombosis, 9th ed: American College of Chest Physicians Evidence-Based Clinical Practice Guidelines. Chest, 2012. 141(2 Suppl): p. e495S-e530S.

[13]. SHANKS, R.M.Q., et al., Heparin Stimulates Staphylococcus aureus Biofilm Formation. Infection and Immunity, 2005. 73(8): p. 4596-4606.

[14]. Moran, J.M.B., et al., A Randomized Trial Comparing Gentamicin/Citrate and Heparin Locks for Central Venous Catheters in Maintenance Hemodialysis Patients. American journal of kidney diseases, 2011. 59(1): p. 102-107.

[15]. Shanks, R.M.Q., et al., Catheter lock solutions influence staphylococcal biofilm formation on abiotic surfaces. Nephrology Dialysis Transplantation, 2006. 21(8): p. 2247-2255.

[16]. Szymańska, J., et al., Locked Away—Prophylaxis and Management of Catheter Related Thrombosis in Hemodialysis. Journal of clinical medicine, 2021. 10(11): p. 2230.

[17]. Hermite, L., et al., Sodium citrate versus saline catheter locks for non-tunneled hemodialysis central venous catheters in critically ill adults: a randomized controlled trial. Intensive Care Med, 2012. 38(2): p. 279-85.

[18]. Quenot, J.P., et al., Trisodium citrate 4% versus heparin as a catheter lock for non-tunneled hemodialysis catheters in critically ill patients: a multicenter, randomized clinical trial. Ann Intensive Care, 2019. 9(1): p. 75.

[19]. Honore, P.M., et al., What should be the best dialysis catheter lock in critically ill patients? Crit Care, 2019. 23(1): p. 339.

[20]. Zhao, Y., et al., Citrate versus heparin lock for hemodialysis catheters: a systematic review and meta-analysis of randomized controlled trials. Am J Kidney Dis, 2014. 63(3): p. 479-90.

[21]. Correa Barcellos, F., et al., Comparative effectiveness of 30 % trisodium citrate and heparin lock solution in preventing infection and dysfunction of hemodialysis catheters: a randomized controlled trial (CITRIM trial). Infection, 2016. 45(2): p. 139-145.

[22]. Pierce, D.A. and M.V. Rocco, Trisodium Citrate: An Alternative to Unfractionated Heparin for Hemodialysis Catheter Dwells. Pharmacotherapy, 2010. 30(11): p. 1150-1158.

[23]. Sheng, K.X., et al., Comparative efficacy and safety of lock solutions for the prevention of catheter-related complications including infectious and bleeding events in adult haemodialysis patients: a systematic review and network meta-analysis. Clin Microbiol Infect, 2020. 26(5): p. 545-552.

[24]. Perez-Granda, M.J., et al., Randomized clinical trial analyzing maintenance of peripheral venous catheters in an internal medicine unit: Heparin vs. saline. PLoS One, 2020. 15(1): p. e0226251.

[25]. Schallom, M.E., et al., Heparin or 0.9% sodium chloride to maintain central venous catheter patency: a randomized trial. Crit Care Med, 2012. 40(6): p. 1820-6.

[26]. Goossens, G.A., et al., Comparing normal saline versus diluted heparin to lock non-valved totally implantable venous access devices in cancer patients: a randomised, non-inferiority, open trial. Ann Oncol, 2013. 24(7): p. 1892-1899.

[27]. Lopez-Briz, E., et al., Heparin versus 0.9% sodium chloride locking for prevention of occlusion in central venous catheters in adults. Cochrane Database Syst Rev, 2018. 7: p. CD008462.

[28]. Landry, D.L., et al., Emergence of gentamicin-resistant bacteremia in hemodialysis patients receiving gentamicin lock catheter prophylaxis. Clin J Am Soc Nephrol, 2010. 5(10): p. 1799-804.

[29]. Dixon, J.J., M. Steele and A.D. Makanjuola, Anti-microbial locks increase the prevalence of Staphylococcus aureus and antibiotic-resistant Enterobacter: observational retrospective cohort study. Nephrol Dial Transplant, 2012. 27(9): p. 3575-81.

[30]. Pittiruti, M., et al., Evidence-based criteria for the choice and the clinical use of the most appropriate lock solutions for central venous catheters (excluding dialysis catheters): a GAVeCeLT consensus. J Vasc Access, 2016. 17(6): p. 453-464.
